# RNA Sequencing of Immune Response-Related Gene Expression Characteristics in Bovine Mammary Glands Infected with *Escherichia coli*

**DOI:** 10.3390/microorganisms13102226

**Published:** 2025-09-23

**Authors:** Kai Zhang, Yuanyuan Zhang, Hong Su, Min Zhang, Feifei Zhao, Daqing Wang, Guifang Cao, Yong Zhang, Caiyun Wang

**Affiliations:** 1 College of Veterinary Medicine, Inner Mongolia Agricultural University, Hohhot 010011, China; zhangkai040423@163.com (K.Z.); zyyworkaccount@163.com (Y.Z.); hongsu1995@126.com (H.S.); zhangmin5400@126.com (M.Z.); imauzff@126.com (F.Z.); wangdaqing050789@126.com (D.W.); zhy1956@263.net (Y.Z.); 2 Animal Embryo and Developmental Engineering Key Laboratory of Higher Education, Institutions of Inner Mongolia Autonomous Region, Hohhot 010011, China; 3 Inner Mongolia Autonomous Region Key Laboratory of Basic Veterinary Medicine, Hohhot 010011, China; 4 College of Life Sciences, Inner Mongolia University, Hohhot 010011, China; guifangcao@126.com

**Keywords:** *Escherichia coli*, Bovine mastitis, infection, RNA sequencing, genes related to immune responses

## Abstract

Bovine mastitis is one of the most prevalent and economically significant diseases affecting dairy cows worldwide, with *Escherichia coli* (*E. coli*) recognized as one of the principal pathogens causing acute mastitis. The innate immune system plays a crucial role in the defense of the bovine mammary gland, serving as the first line of defense against pathogen invasion. This study elucidated the pathological mechanisms and immune response-related molecular regulatory networks involved in *E. coli*-induced bovine mastitis. Histopathological and apoptosis analyses of mammary tissues were performed using hematoxylin-eosin (HE) staining and TUNEL staining, respectively, while RNA sequencing (RNA-seq) was conducted to identify differentially expressed genes (DEGs) and their associated signaling pathways. HE staining revealed typical inflammatory lesions in the mammary glands of mastitis cows. TUNEL staining further confirmed that the level of apoptosis in the mastitis group was significantly higher than in the healthy control group (*p* < 0.0001). RNA-seq analysis identified 2717 DEGs, with 2238 upregulated and 479 downregulated genes. The top 20 significantly upregulated genes (e.g., *S100A12*, *IL1RN*, *IL1R2*, *CXCL8*, *SAA3*, *S100A8*, *S100A9*, *TREML2*, *TREM1*, *M-SAA3.2*, *PTX3*, *MMP9*) were predominantly involved in inflammatory immune regulation, acute phase responses (e.g., HP, SAA3), and cellular signal transduction (e.g., *PLEK*, *LPAR3*). Gene Ontology (GO) enrichment analysis revealed that these DEGs were mainly associated with biological processes, such as signal transduction, immune response, inflammatory response, and transcriptional regulation. Kyoto Encyclopedia of Genes and Genomes (KEGG) pathway analysis indicated that these DEGs were significantly enriched in key inflammatory and immune regulatory pathways, including the TNF signaling pathway, C-type lectin receptor signaling pathway, Chemokine signaling pathway, NOD-like receptor signaling pathway, NF-κ B signaling pathway, and IL-17 signaling pathway, suggesting that these pathways play central roles in the mammary immune defense against *E. coli* infection. In conclusion, this study demonstrated at the histopathological, cellular apoptosis, and transcriptomic levels that *E. coli* infection induces mammary tissue damage and apoptosis by activating immune and inflammation-related genes (*S100A12*, *IL1RN*, *IL1R2*, *CXCL8*, *SAA3*, *S100A8*, *S100A9*, *TREML2*, *TREM1*, *M-SAA3.2*, *PTX3*, *MMP9*) and key signaling pathways (TNF signaling pathway, C-type lectin receptor signaling pathway, Chemokine signaling pathway, NOD-like receptor signaling pathway, NF-κ B signaling pathway, IL-17 signaling pathway). The findings of this study provide a theoretical basis for probing into the pathogenesis of bovine mastitis and the development of targeted interventions.

## 1. Introduction

Bovine mastitis is an inflammatory condition of the mammary gland triggered by various external factors, causing the greatest economic losses to the dairy industry worldwide. The rising incidence of mastitis in dairy farming not only adversely impacts economic returns but also compromises animal health and welfare, potentially leading to the culling of affected cows in severe cases [1]. Despite the modernization and industrial-scale management of dairy farms and notable improvements in veterinary services and herd management, bovine mastitis remains one of the leading causes of economic losses in the global dairy sector [2].

Among the different types of mastitis, bacterial mastitis is the most prevalent form [3]. Over 150 microbial pathogens have been identified as causative agents of bovine mastitis, with *Escherichia coli* (*E. coli*) recognized as a primary pathogen responsible for acute mastitis in cows. In severe cases, *E. coli*-induced mastitis can lead to systemic clinical complications, such as sepsis [4,5]. *E. coli* infections may also cause subclinical mastitis with persistent pathological alterations [6].

In dairy farm management, antibiotics are frequently employed to control mastitis. However, uncontrolled prescription of antibiotics can exacerbate antimicrobial resistance, leading to treatment failures [7]. Beyond the infection or antibiotic use, the host immune response often plays a more decisive role in determining disease severity. As the first line of defense against invading pathogens, the innate immune system is pivotal [8]. Unlike acquired immunity, innate immunity provides a rapid and nonspecific response during the early stages of infection. The innate immune defense of the bovine mammary gland comprises multiple components, including physical barriers, immune cells, and soluble immune factors, which cooperate to establish a complex defense network. Most intramammary infections are initiated when bacteria overcome the physical barrier of the teat canal. Once pathogens enter the teat cistern, inadequate innate immune responses allow bacterial proliferation and colonization within the mammary tissue [9]. The immune capacity of the mammary gland is often diminished during the periparturient period and early lactation. Hence, breeding cows with enhanced resistance to mastitis necessitates an in-depth elucidation of the mechanisms underlying mammary immune responses [10].

Earlier studies utilizing conventional immunological and molecular biology methods have provided valuable insights into the immune responses elicited by *E. coli* infections in the bovine mammary gland, revealing phenomena such as the release of pro-inflammatory cytokines and the recruitment of immune cells [10]. However, these investigations have largely focused on individual or a limited number of genes and proteins, lacking a comprehensive understanding of the molecular mechanisms underlying the immune response to *E. coli* infection. The advent of high-throughput sequencing technologies has propelled the development of RNA-seq, offering powerful tools for exploring complex biological processes at the genome-wide level. RNA-seq enables holistic examination of the expression patterns of all intracellular RNA transcripts, facilitating systematic elucidation of gene regulatory networks and the discovery of novel functional genes and signaling pathways [11]. However, despite recent advances in molecular biology and immunology, the molecular mechanisms and roles of numerous genes involved in the innate immune response to bacterial infection in the bovine mammary gland remain obscure.

Therefore, this study focused on *E. coli*-induced bovine mastitis, integrating histopathological assessment, apoptosis detection, and RNA-seq to characterize gene expression changes. Differentially expressed genes (DEGs) were identified and subjected to functional categorization and enrichment analyses, with particular emphasis on key genes and signaling pathways associated with immune responses. By delving into the expression features of immune response-related genes during *E. coli*-induced mastitis, this study provides novel insights and theoretical foundations for understanding the molecular pathogenesis of bovine mastitis.

## 2. Materials and Methods

### 2.1. Reagents and Chemicals

Physiological saline (Hyclon, Logen, UT, USA); 4% paraformaldehyde, HE staining kit (Solarbio, Beijing, China); TUNEL Alexa Fluor 488 Apoptosis Detection Kit (Arcegen, Delaware, NJ, USA); Trizol (Thermo Fisher, Waltham, MA, USA); Axy Prep Multisource Total mRNA Miniprep Kit (Axygen Scientific, Union City, CA, USA); Primer Script RT Master Mix (Takara, Shiga, Japan); SYBR Green Master (Rox) (Roche, Basel, Switzerland).

### 2.2. Sample Collection

This study selected 15 lactating adult Holstein cows at a slaughterhouse in Hohhot and harvested their mammary tissues within 30 min post-slaughter. Partial samples were fixed in 4% paraformaldehyde for histopathological examination, while the remaining samples were preserved in liquid nitrogen for subsequent analyses. The external appearance of the mammary glands was visually inspected. The pathological damage and apoptotic characteristics of mammary tissue were analyzed using hematoxylin-eosin (HE) staining and TUNEL staining techniques. Moreover, bacteriological examination of the bovine mammary gland was performed. Based on these evaluations, three healthy mammary gland samples and three *E. coli*-induced mastitic mammary gland samples were selected for subsequent analysis.

### 2.3. HE Staining

Mammary tissue samples were fixed in 4% paraformaldehyde at room temperature for at least 24 h, then rinsed under running tap water for 40 min to remove residual fixative. The samples were processed through a graded ethanol series: 70% ethanol for 40 min (once), 95% ethanol twice for 20 min each, and 100% ethanol twice for 20 min each. Tissue transparency was achieved by immersing samples in xylene twice for 20 min each. Molten paraffin was poured into embedding molds, and the tissues were positioned until solidification (approximately 15–20 min). The paraffin-embedded blocks were sectioned at approximately 4 μm thickness, floated on 37 °C deionized water, mounted onto glass slides, and dried in a 65 °C oven for 15 min, followed by further drying at 38 °C. Subsequently, the sections were immersed in xylene twice for 10 min each, then rehydrated sequentially in 100% ethanol twice (5 min each), followed by sequential immersions in 95% ethanol (5 min), 70% ethanol (2 min), and a final rinse with distilled water. Sections were stained with hematoxylin for 8 min, rinsed under running water for 5 min, differentiated with 1% hydrochloric acid in ethanol for 30 s, and rinsed again with water for 1 min. Bluing was performed using 0.2% ammonia water for 1 min, followed by washing with water for 3–5 min. After washing with 95% ethanol several times, the sections were then counterstained with eosin for 2 min. Dehydration was carried out by sequential immersion in 80%, 90%, 95%, and twice in 100% ethanol for 5 min each. Finally, sections were cleared in xylene I and II for 5 min each, mounted with neutral balsam, and examined under a light microscope.

### 2.4. TUNEL Staining

Paraffin sections were first deparaffinized with xylene twice for 10 min each, then rehydrated sequentially in 100% ethanol twice (5 min each), 95% ethanol (2 min), 70% ethanol (2 min), and rinsed with distilled water. Next, the sections underwent PBS washing three times for 5 min each. Proteinase K working solution was applied at 37 °C for 30 min, followed by PBS washes (three times, 5 min each). Endogenous peroxidase activity was blocked by treatment with H_2_O_2_ at room temperature for 10 min, then sections were washed with PBS three times for 5 min each. The Terminal Deoxynucleotidyl Transferase (TdT) enzyme reaction mixture was applied at 37 °C in the dark for 60 min, followed by PBS washes (three times, 5 min each). Streptavidin-HRP solution was added and incubated at 37 °C for 30 min in the dark, then washed with PBS three times for 5 min each. Diaminobenzidine (DAB) substrate was used for color development, followed by additional PBS washes (three times, 5 min each). Then, sections were counterstained with hematoxylin, mounted, and examined microscopically. Analysis of positive signals was performed using ImageJ 1.48v software.

### 2.5. RNA-Seq Analysis

Total RNA was extracted using Trizol reagent following the manufacturer′s procedure. Sequencing was performed using the Illumina Novaseq 6000 platform with pair-end 150-base reads. LC Bio Technology CO., Ltd. (Hangzhou, China) sequenced the cDNA libraries on the Illumina sequencing platform. Raw data filter and mapping were described in the ref. [12]. The gene expression level was further normalized by using the fragments per kilobase of transcript per million (FPKM) mapped reads method to eliminate the influence of different gene lengths and the amount of sequencing data on the calculation of gene expression. Differentially expressed genes (DEGs) were identified using the edge R package (accessed on 10 March 2025 http://www.r-project.org/, version 3.6) across samples with fold changes ≥2 and a false discovery rate-adjusted *p* (*q*-value) < 0.05. DEGs were then subjected to an enrichment analysis of hierarchical clustering, Gene Ontology (GO) function and Genes and Genomes (KEGG) pathways, and *q*-values < 0.05 were used as a threshold.

### 2.6. Real-Time-PCR Analysis

RNA was isolated from the mammary tissues of healthy dairy cows and those with mastitis caused by *E*. *coli* infection. Total mRNA extraction and reverse transcription were performed. The polymerase chain reaction conditions were as follows: 50 °C, 2 min; 95 °C, 10 min; 95 °C, 15 s; 60 °C, 60 s; for 40 cycles. The annealing temperature was 58 °C. The primers used in this study are listed in Table 1. The results were calculated using the 2^−∆∆Ct^ calculation method.

### 2.7. Statistical Analysis

In this experiment, the linear association strength between the mammary tissues of normal dairy cows and those of cows with mastitis was evaluated using the Pearson correlation coefficient among the samples and the R software (Version 3.6) for correlation analysis. All data had been analyzed with the use of GraphPad Prism 8 and are expressed as the mean ± standard deviation (SD). Statistical significance was evaluated by one-way analysis of variance (ANOVA) followed by Tukey′s multiple comparisons test or two-way ANOVA (Bonferroni′s post-test), as appropriate. Differences with *p* values ≤ 0.05 were considered statistically significant (ns *p* > 0.05, * *p* < 0.05, ** *p* < 0.01, *** *p* < 0.001, and **** *p* < 0.0001 compared to the normal group).

## 3. Results

### 3.1. HE Staining Results

**Figure 1:**
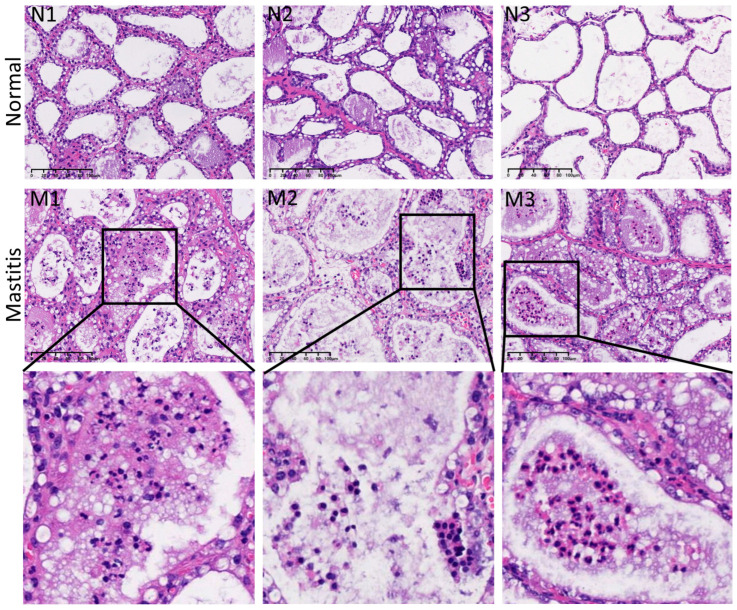
HE staining results of bovine mammary gland tissues. N1–N3 represent healthy lactating mammary gland samples, and M1–M3 are mammary gland samples with *E. coli*-induced mastitis. The black box shows the infiltration of inflammatory cells in the breast tissue sample. The scale bar is 100 μm.

As shown in Figure 1 (N1–N3), HE staining of healthy mammary tissue revealed a well-organized structure without apparent damage, displaying intact mammary alveolar architecture with clearly defined interstitial spaces. The mammary gland exhibited densely arranged, round or oval alveoli lined by a continuous monolayer of cuboidal epithelial cells. Dense collagen fibers separated the lobules within the stroma, adipocytes were scattered throughout, and vascular structures remained intact. No infiltration of immune cells, such as neutrophils or lymphocytes, was observed in the alveolar lumen, which is consistent with the morphological characteristics of healthy lactating mammary glands.

In contrast, HE staining of *E. coli*-infected mastitic tissues (Figure 1 M1–M3) displayed abundant infiltration of immune cells, predominantly neutrophils and lymphocytes, within the alveolar lumen. Partial necrosis and exfoliation of epithelial cells were observed in the stroma, accompanied by erythrocyte extravasation. These findings indicate that the mammary tissues from cows with mastitis exhibited typical histopathological features of bacterial mastitis.

### 3.2. Detection of Apoptosis in Mastitic Mammary Glands by TUNEL Staining

**Figure 2:**
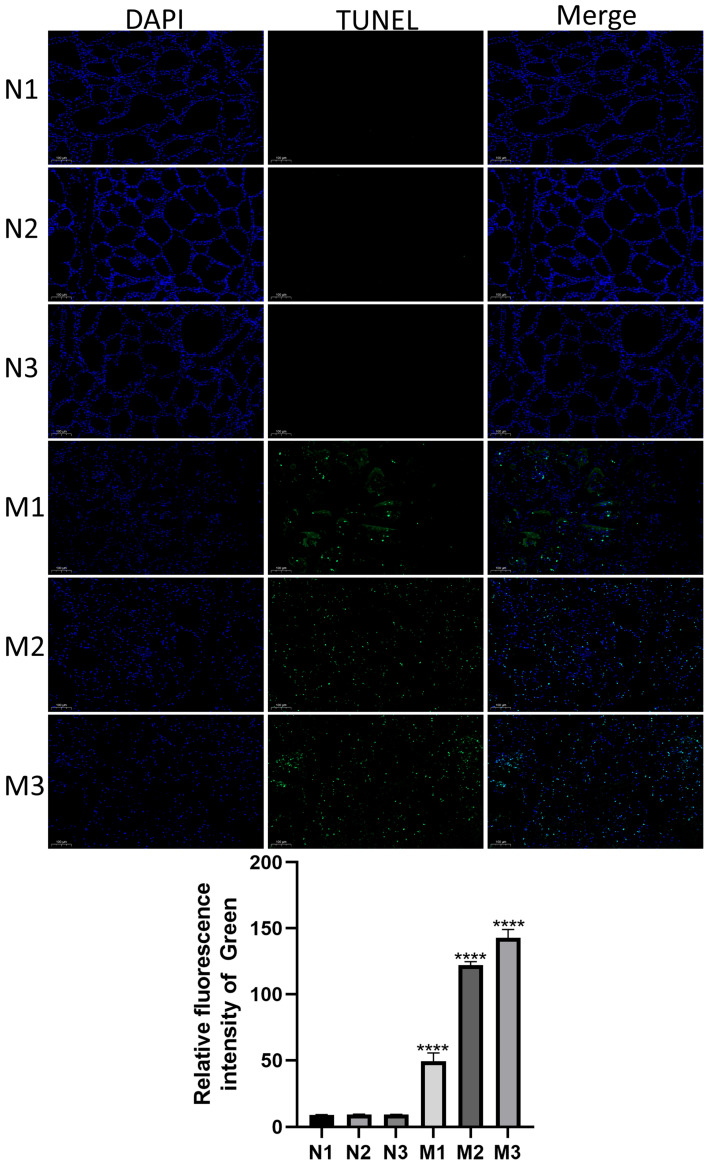
Apoptosis in bovine mammary glands during mastitis. N1–N3 represent healthy lactating mammary gland samples, and M1–M3 are mammary gland samples with *E. coli*-induced mastitis. The cell nucleus is stained blue, and apoptotic cells are stained green. The scale bar is 100 μm. * denotes a statistical difference between groups, with *p* ≤ 0.05 considered significant. **** represents *p* < 0.0001.

To assess apoptosis levels in bovine mammary tissues during mastitis, TUNEL staining was performed on samples from the healthy (Normal) and mastitic (Mastitis) groups. As illustrated in Figure 2, a significant increase in apoptotic cells was observed in the Mastitis group (M1, M2, M3) compared with the Normal group (N1, N2, N3) (*p* < 0.0001). These results suggest that *E. coli*-induced mastitis markedly elevated mammary epithelial cell apoptosis.

### 3.3. Assessment of Sample Correlation and DEG Analysis

**Figure 3:**
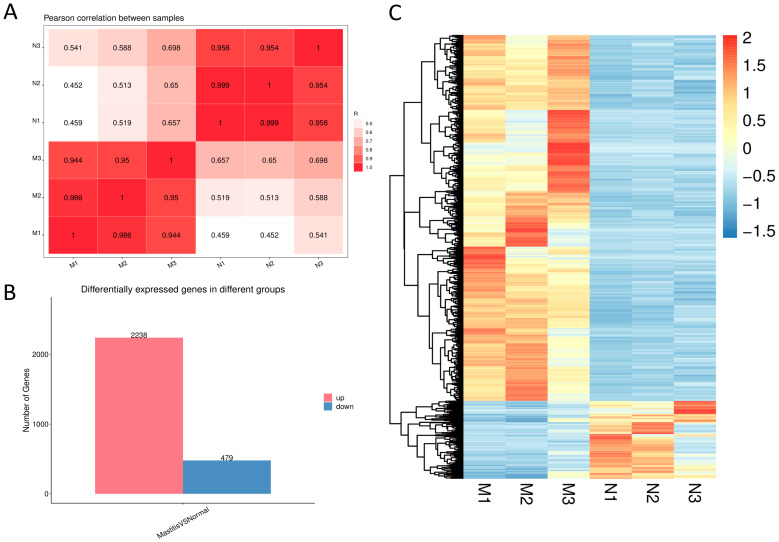
Correlation Evaluation and DEG Analysis between Samples. (**A**) shows the result of the Pearson correlation analysis. (**B**) presents a bar chart showing the quantities of up-regulated genes and down-regulated genes. (**C**) shows the hierarchical clustering analysis result of DEGs.

Pearson correlation analysis was conducted to evaluate the reproducibility and consistency among biological replicates, facilitating the identification and exclusion of potential outlier samples. As shown in Figure 3A, the Pearson correlation coefficients (R) for all biological replicates exceeded 0.944, indicating strong inter-sample correlation suitable for subsequent data analyses. DEGs between the Normal and Mastitis groups were identified, with the number of upregulated and downregulated genes summarized in a bar chart and their distribution visualized in a hierarchical clustering heatmap. As illustrated in Figure 3B,C, 2238 genes were significantly upregulated and 479 were significantly downregulated in the Mastitis group compared to the Normal group. Hierarchical clustering of DEGs revealed distinct gene expression profiles between the Normal and Mastitis groups (Figure 3C), demonstrating that *E. coli* challenge induced substantial transcriptional reprogramming in bovine mammary tissues. These DEGs likely represent candidate genes involved in the response to mastitis pathogenesis.

### 3.4. DEG Screening

To identify the most significantly altered genes, the top 20 upregulated DEGs were subjected to in-depth analysis. As shown in Table 2 after excluding unannotated entries in the database, the markedly upregulated DEGs included *S100A12*, *IL1RN*, *CXCL8*, *SAA3*, *S100A9*, *PLEK*, *IL1R2*, *LPAR3*, *TREML2*, *M-SAA3.2*, *S100A8*, *MYBPH*, *SERPINF2*, *PTX3*, *HP*, *MMP9*, *TCN1*, and *TREM1*. Through further functional classification, genes associated with inflammation and immune regulation include *S100A12*, *IL1RN*, *IL1R2*, *CXCL8*, *SAA3*, *S100A8*, *S100A9*, *TREML2*, *TREM1*, *M-SAA3.2*, *PTX3*, and *MMP9*; genes related to acute phase response and stress encompass *SAA3*, *HP*, and *SERPINF2*; genes involved in cellular signal transduction consist of *PLEK* and *LPAR3*. These findings indicate that upon *E. coli* stimulation of the bovine mammary gland, these DEGs predominantly participate in inflammatory and immune regulatory responses.

### 3.5. GO Enrichment Analysis

**Figure 4:**
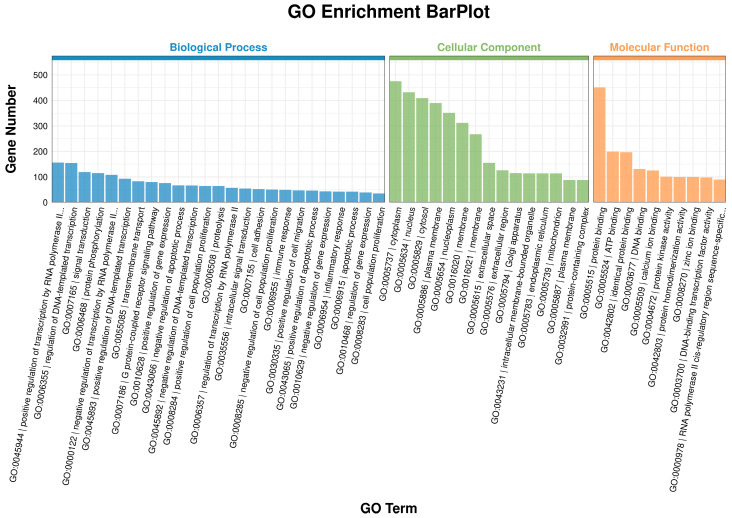
GO enrichment analysis results. GO:0000978: RNA polymerase II cis-regulatory region sequence-specific DNA binding.

GO enrichment analysis was performed across Biological Process (BP), Cellular Component (CC), and Molecular Function (MF) categories. The results are presented in Figure 4. BP analysis revealed that, compared to the Normal group, DEGs in the Mastitis group were significantly enriched in processes such as Positive regulation of transcription by RNA polymerase II, Regulation of DNA-templated transcription, Signal transduction, Immune response, and Inflammatory response. CC analysis indicated that DEGs in the Mastitis group were mainly localized to the cytoplasm, nucleus, and cytosol, which represented the top three enriched categories. MF analysis showed that DEGs in the Mastitis group were primarily enriched in protein binding, ATP binding, and DNA binding.

These results suggest that mastitis induces specific transcriptional programs in mammary tissue, involving signal transduction cascades and molecular functions such as protein/DNA/ATP binding, altering gene expression and cellular activity, and ultimately manifesting as the phenotypic characteristics of bovine mastitis.

### 3.6. KEGG Enrichment Analysis

**Figure 5:**
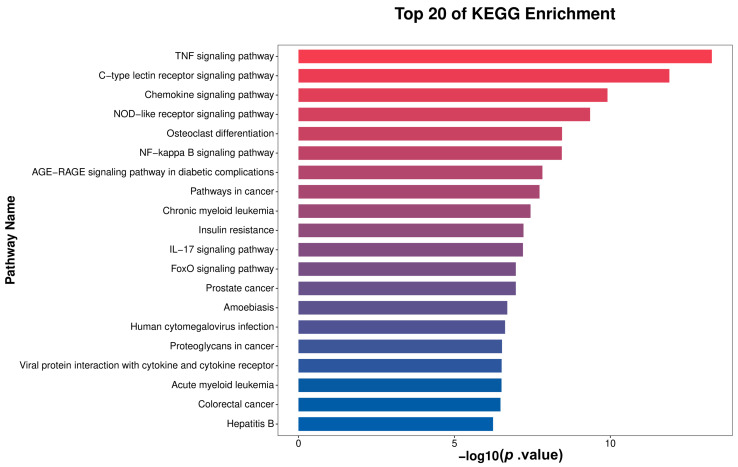
KEGG enrichment analysis results.

The top 20 enriched pathways identified through KEGG analysis were visualized in Figure 5 as a bar plot. Significantly enriched pathways include the TNF signaling pathway, C-type lectin receptor signaling pathway, Chemokine signaling pathway, NOD-like receptor signaling pathway, NF-κ B signaling pathway, and IL-17 signaling pathway. These pathways are closely associated with immune and inflammatory responses, indicating that the DEGs between the Mastitis and Normal groups are significantly involved in immune-inflammatory regulatory processes relevant to mastitis. Collectively, these pathways constitute a coordinated immune-inflammatory regulatory network underlying the onset and progression of mastitis. These results demonstrate that mastitis involves the synergistic activation of immune-inflammatory pathways, with the identified DEGs driving disease development through their participation in these pathways, providing insights into subsequent analysis of underlying mechanisms and development of intervention strategies.

### 3.7. Validation of DEGs

**Figure 6:**
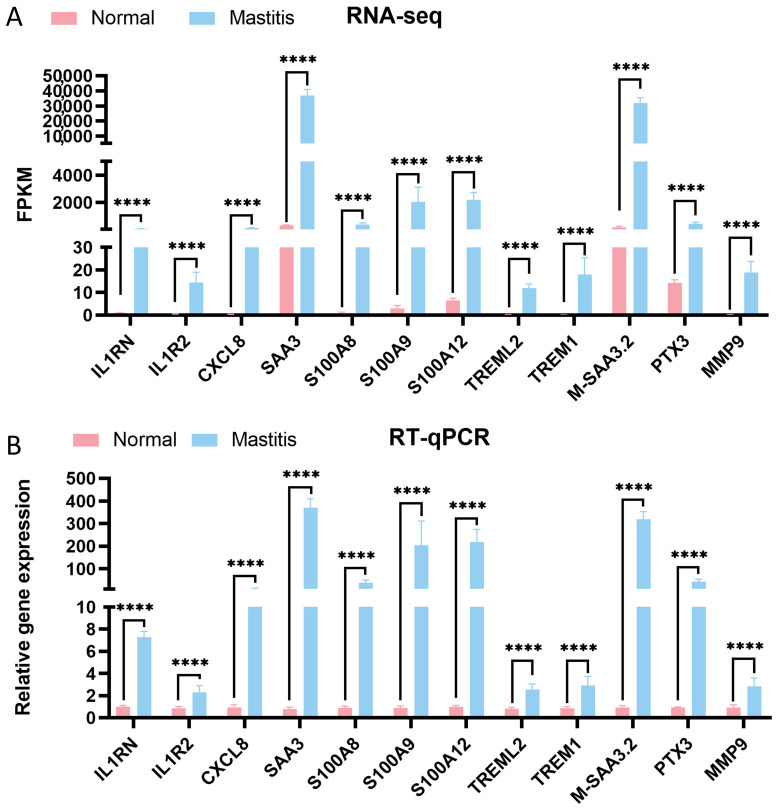
Validation of DEGs. (**A**) shows the results of RNA-seq, and (**B**) shows the results of RT-qPCR. * denotes a statistical difference between groups, with *p* ≤ 0.05 considered significant. **** represents *p* < 0.0001.

To validate the reliability of the RNA-seq results, genes associated with inflammation and immune regulation (*S100A12*, *IL1RN*, *IL1R2*, *CXCL8*, *SAA3*, *S100A8*, *S100A9*, *TREML2*, *TREM1*, *M-SAA3.2*, *PTX3*, and *MMP9*) were selected from the top 20 upregulated DEGs. RT-qPCR was used to assess their expression levels. As shown in Figure 6, compared to the Normal group, the expression of these genes was significantly elevated in the Mastitis group (*p* < 0.0001). This is consistent with the RNA-seq results, thereby confirming the robustness of the transcriptomic data and laying a solid foundation for subsequent research.

## 4. Discussion

Bovine mastitis remains one of the principal diseases impacting the global dairy industry [1], with *E. coli* recognized as a leading pathogen causing acute mastitis that, in severe cases, can result in systemic clinical manifestations, such as sepsis [4,5]. This study integrated histopathological analysis, apoptosis detection, and transcriptomic profiling to delve into the pathological features and molecular mechanisms underlying *E. coli*-induced bovine mastitis, thereby providing crucial experimental evidence for elucidating the pathogenesis of this disease.

First, HE staining illustrated striking pathological differences between healthy mammary glands from lactating cows and those infected with *E. coli*. Healthy mammary tissues exhibited intact alveolar structures, clear stroma, and absence of immune cell infiltration—hallmarks of normal mammary architecture. In contrast, mastitic tissues displayed classic features of acute bacterial mastitis, including massive infiltration of neutrophils and lymphocytes into the alveolar lumen, necrosis and desquamation of interstitial epithelial cells, and erythrocyte extravasation within the stroma. These pathological alterations directly reflect the intense inflammatory response and tissue destruction triggered by pathogen invasion.

Furthermore, TUNEL staining demonstrated markedly elevated levels of apoptosis in mastitic tissues compared to healthy controls (*p* < 0.0001). This indicates that, beyond direct bacterial damage and necrosis mediated by inflammatory factors, death receptor activation or mitochondrial dysfunction within the inflammatory microenvironment may contribute to mammary epithelial cell apoptosis. Moreover, this process may also be linked to TNF-α-induced apoptosis mechanisms [12].

RNA-seq revealed substantial alterations in gene expression profiles during mastitis. This study identified a large number of DEGs, including 2238 significantly upregulated and 479 downregulated, indicating that *E. coli* infection profoundly impacts transcriptional regulation in mammary tissues. Functional classification of the top 20 significantly upregulated DEGs demonstrated that most are closely related to inflammation and immune modulation (e.g., *S100A12*, *IL1RN*, *IL1R2*, *CXCL8*, *SAA3*, *S100A8*, *S100A9*, *TREML2*, *TREM1*, *M-SAA3.2*, *PTX3*, and *MMP9*). *CXCL8* (IL-8) is a potent cytokine-induced neutrophil chemoattractant [13]. Its upregulation directly corresponds to the massive neutrophil infiltration observed in HE-stained mastitic tissues. The upregulation of IL1RN (IL-1 receptor antagonist) likely represents a negative feedback mechanism in the bovine mammary gland aimed at limiting excessive IL-1-mediated inflammation [14], consistent with findings in pediatric human studies by Ivovona et al. *IL1R2* (Interleukin-1 receptor 2) binds and neutralizes IL-1, thereby preventing excessive inflammatory responses [15]. Members of the S100 protein family—*S100A8*, *S100A9*, and *S100A12*—are pivotal endogenous damage-associated molecular patterns (DAMPs) released by immune cells or damaged epithelial cells, exhibiting potent pro-inflammatory and chemotactic activities [16]. This suggests that in the context of mastitis, upregulation of *S100A8*, *S100A9*, and *S100A12* enhances the production of pro-inflammatory cytokines and Chemokines to maintain host homeostasis. Acute-phase proteins such as *SAA3*, *M-SAA3.2*, *HP*, and *PTX3* are also upregulated, reflecting the localized expression of systemic responses to infection and tissue damage. These proteins mainly participate in opsonization, immune regulation, and tissue repair [17]. Upregulated *TREM1* and *TREML2* (members of the triggering receptor expressed on myeloid cells family, magnifying inflammatory signals) [18], PLEK (involved in leukocyte signal transduction) [19], and *LPAR3* (a lysophosphatidic acid receptor participating in diverse cellular processes) [20,21], together form a complex network for sensing and amplifying inflammatory signals. Meanwhile, the upregulation of *MMP9* (matrix metalloproteinase-9), a molecule involved in tissue remodeling, indicates concurrent tissue degradation and remodeling during inflammation. *MMP9* expression is significantly positively correlated with somatic cell count in milk, aligning with Mao et al.′s findings in bovine mammary epithelial cells [22].

GO enrichment analysis further validated that the core functions of DEGs were centered on biological processes such as immune response, inflammatory response, signal transduction, and transcriptional regulation, particularly positive regulation of transcription by RNA polymerase II. KEGG pathway enrichment analysis mapped key signaling networks underlying mastitis pathogenesis. Among the top 20 significantly enriched pathways, many were directly related to immune inflammatory responses, including innate immune recognition and signaling pathways (e.g., the NOD-like receptor and C-type lectin receptor pathways), which are crucial for recognizing pathogen-associated molecular patterns (PAMPs, e.g., *E. coli* LPS, peptidoglycan) and DAMPs [22]. In this study, the enriched primary inflammatory signaling pathways among DEGs included the TNF signaling pathway, NF-κ B signaling pathway, and IL-17 signaling pathway. These pathways serve as central hubs orchestrating the production of pro-inflammatory cytokines (TNF-α, IL-1β, IL-17), amplifying inflammatory responses, and inducing downstream effector gene expression [23,24]. KEGG pathway analysis indicated significant enrichment in pathways related to chemotaxis and cell migration, such as the Chemokine signaling pathway, which directly regulates the recruitment of neutrophils and lymphocytes to the infection site. This agrees with the histologically observed cellular infiltration in this study [25].

Notably, these pathways do not function in isolation; rather, they intersect and cooperate, forming a highly interconnected immune-inflammatory regulatory network [13]. For instance, activation of NOD-like receptors or TLRs triggers the NF-κ B pathway, which induces expression of genes encoding TNF-α, IL-1β, IL-8, and SAA, while TNF-α signaling can further activate NF-κ B, thus establishing a positive feedback loop to amplify inflammation [13]. The IL-17 pathway can also synergize with TNF signaling to enhance inflammatory responses [24]. Among the identified DEGs in this study, many (such as *CXCL8*, *S100A8/A9*, *PTX3*, and *MMP9*) are key downstream effectors of these pathways. Consequently, mastitis is fundamentally driven by pathogen-triggered cascade activation of an innate immune signaling network centered on NF-κ B, TNF, NLR, and CLR pathways, leading to massive expression of pro-inflammatory cytokines, Chemokines, and acute-phase proteins. This culminates in pronounced neutrophil infiltration, tissue injury (including increased apoptosis), and acute-phase responses.

Finally, RT-qPCR validation of 12 key inflammation/immune-related DEGs (*S100A12*, *IL1RN*, *IL1R2*, *CXCL8*, *SAA3*, *S100A8*, *S100A9*, *TREML2*, *TREM1*, *M-SAA3.2*, *PTX3*, and *MMP9*) confirmed their significant upregulation in mastitic tissues (*p* < 0.0001). This is consistent with RNA-seq data, thereby affirming the reliability of the transcriptomic findings. These validated genes are not only potential biomarkers for mastitis diagnosis but also promising therapeutic targets. For instance, targeting TREM1, inhibiting *S100A8*/*A9* activity, or blocking key inflammatory pathways (e.g., NF-κ B) or their downstream effectors (e.g., *CXCL8*, *MMP9*) could represent novel strategies to mitigate excessive inflammation and tissue damage in mastitis, providing a theoretical basis for clinical management and breeding of disease-resistant dairy cows.

## 5. Conclusions

In summary, this study elucidated that the key upregulated genes (such as *CXCL8*, *S100A8/A9/A12*, *TREM1*, *PTX3*, and *MMP9*) were found to collaboratively drive neutrophil chemotaxis, amplification of pro-inflammatory signaling, and tissue remodeling. These DEGs were significantly enriched in pathways related to innate immune recognition (NOD-like and C-type lectin receptor signaling), inflammatory hubs (NF-κ B, TNF, IL-17 signaling), and chemotaxis and migration pathways. Through positive feedback loops, these pathways trigger explosive expression of pro-inflammatory cytokines and DAMPs, leading to a cascade amplification of inflammation and consequent tissue injury. The identification of these core genes provides potential biomarkers for early diagnosis of mastitis and probably highlights promising therapeutic targets for intervention.

## Figures and Tables

**Table 1:** Primer sequences for RT-qPCR.

Gene Name	Sequences (5′-3′)	Accession Number
*β-actin*	F:5′-CCAAGGCCAACCGTGAGAAGAT-3′ R:5′-CCACGTTCCGTGAGGATCTTCA-3′	NM_173979.3
*IL1RN*	F:5′-AGAGGCCGTGAACATCACTG-3′ R:5′-CCTCCAGTGATGTGCAGAGG-3′	NM_174357.3
*IL1R2*	F: 5′-CCAGGCTGACAATCCCATGT-3′ R:5′-AAGGTGTTGTTGGCTGTCCA-3	NM_001046210.2
*CXCL8*	F: 5′-GCTGGCTGTTGCTCTCTTGG-3′ R: 5′-GGGTGGAAAGGTGTGGAATGTG-3′	NM_173925.2
*SAA3*	F: 5′-AAACTATGACGCTGCCCGAA-3′ R:5′-CCCATTCGTTGGCAAACTGG-3′	NM_181016.3
*S100A8*	F: 5′-TGTGCCATTAACTCCCTGATTGAC-3′ R: 5′-TTGAACCAAGTGTCCGCATCC-3′	NM_001113725.2
*S100A9*	F: 5′-ATCATGGAGGATCTGGACACAAATG-3′ R: 5′-CGTGGGAGGCTACCGTCAG-3′	NM_001046328.2
*S100A12*	F: 5′-GCTGGAAGATCACCTGGAGG-3′ R:5′-GCTTCAGCTCACGCTTGTTG-3′	NM_174651.3
*TREML2*	F: 5′-CAGCTGGACTCCTCACCTTG-3′ R:5′-AGTGCTGGAGAAACTGGAGC-3′	XM_015459834.3
*TREM1*	F: 5′-ACATCCCCAGTGAAGGCATG-3′ R:5′-GGACAGGGTGGAACAGGATG-3′	NM_206970.1
*M-SAA3.2*	F: 5′-ACCTTTCCACGGGCATCATT-3′ R:5′-CGCGGGCATGGAAGTATTTG-3′	NM_001242573.1
*PTX3*	F: 5′-CGGCGGAGAACTCAGATGATTATG-3′ R: 5′-CCAGCATGGTGAAGAGCTTGTC-3′	NM_001076259.2
*MMP9*	F: 5′-GGTGCTGGCTTGCTGCTCTG-3′ R: 5′-TTGGTGAGGTTGGTTCGTGGTTC-3′	NM_174744.2

**Table 2:** Top 18 upregulated DEGs.

Gene ID	Gene Name
ENSBTAG00000012638	*S100A12*
ENSBTAG00000019665	*IL1RN*
ENSBTAG00000019761	*CXCL8*
ENSBTAG00000049589	*SAA3*
ENSBTAG00000006505	*S100A9*
ENSBTAG00000009658	*PLEK*
ENSBTAG00000006343	*IL1R2*
ENSBTAG00000003791	*LPAR3*
ENSBTAG00000015707	*TREML2*
ENSBTAG00000054278	*M-SAA3.2*
ENSBTAG00000012640	*S100A8*
ENSBTAG00000011465	*MYBPH*
ENSBTAG00000020859	*SERPINF2*
ENSBTAG00000009012	*PTX3*
ENSBTAG00000006354	*HP*
ENSBTAG00000020676	*MMP9*
ENSBTAG00000020580	*TCN1*
ENSBTAG00000017593	*TREM1*

## Data Availability

The original contributions presented in this study are included in the article. Further inquiries can be directed to the corresponding author.

## Abbreviations

The following abbreviations are used in this manuscript:

*E. coli*	*Escherichia coli*
HE	Hematoxylin-eosin
RNA-seq	RNA sequencing
DEGs	Differentially expressed genes
GO	Gene Ontology
KEGG	Kyoto Encyclopedia of Genes and Genomes
BP	Biological Process
CC	Cellular Component
MF	Molecular Function
TdT	Terminal Deoxynucleotidyl Transferase
DAB	Diaminobenzidine
